# *In vitro* and *in vivo* combination of cepharanthine with anti-malarial drugs

**DOI:** 10.1186/1475-2875-13-90

**Published:** 2014-03-12

**Authors:** Camille Desgrouas, Jérôme Dormoi, Charles Chapus, Evelyne Ollivier, Daniel Parzy, Nicolas Taudon

**Affiliations:** 1 UMR-MD3, Laboratoire de Pharmacognosie et Ethnopharmacologie, Faculté de Pharmacie, Aix-Marseille Université, 27 boulevard Jean Moulin, CS30064, 13385 Marseille cedex 5, France; 2 Institut de Recherche Biomédicale des Armées, Unité de parasitologie, BP73, 91223 Brétigny-sur-Orge, France; 3 Institut de Recherche Biomédicale des Armées, Unité de biologie moléculaire, BP73, 91223 Brétigny-sur-Orge, France; 4 UMR – MD3, Faculté de pharmacie, Aix-Marseille-Université, Marseille, France; 5 Institut de Recherche Biomédicale des Armées, Unité de toxicologie analytique, BP73, 91223 Brétigny-sur-Orge, France

**Keywords:** Malaria, Cepharanthine, Antimalarial drug combinations, Isobolograms, *in vivo*

## Abstract

**Background:**

*Stephania rotunda* is used by traditional health practitioners in Southeast Asia to treat a wide range of diseases and particularly symptoms related to malaria. Cepharanthine (CEP) is an alkaloid isolated from this plant with potential innovative antiplasmodial activity. The analysis of interactions between antiplasmodial drugs is necessary to develop new drugs combinations to prevent *de novo* emergence of resistance. The objective of this study was to evaluate the anti-malarial activity of CEP in combination with usual anti-malarial compounds, both *in vitro* and *in vivo*.

**Methods:**

A fixed ratio method using the isotopic micro test was performed on the chloroquine-resistant plasmodial strain W2 to build isobolograms from eight CEP-based combinations with standard anti-malarial drugs. The efficacy of two combinations was then evaluated in the BALB/c mouse infected with *Plasmodium berghei* ANKA strain.

**Results:**

*In vitro*, efficiency gains were observed when CEP was combined with chloroquine (CQ), lumefantrine (LUM), atovaquone (ATO), piperaquine (PPQ) and particularly monodesethylamodiaquine (MdAQ), whereas an antagonistic interaction was observed with dihydroartemisinin (DHA) and mefloquine (MQ). *In vivo*, the combination of CEP with CQ or amodiaquine (AQ) improved significantly the survival of mice and extended the delay for parasitic recrudescence.

**Conclusion:**

All these observations suggest that CEP could be an interesting lead compound in the development of a combination therapy against malaria.

## Background

Affecting about 216 million and killing 660,000 people in 2010, malaria remains one of the most widespread parasitic tropical diseases [1]. The spread of resistance of *Plasmodium falciparum* to most anti-malarial drugs is an important difficulty in the treatment of this disease. The use of artemisinin combination therapy (ACT) as first-line treatment of uncomplicated malaria caused by *P. falciparum* was officially recommended by the WHO in 2006 [2]. Unfortunately, the emergence of resistance to artemisinin derivatives was recently reported from the Southeast Asia [3,4]. In this context, new anti-malarial compounds are urgently needed to treat this major endemic disease. Molecules structurally different from the available anti-malarial drugs and targeting innovative and independent metabolism pathways are particularly asked for. Drawing from the rich plant biodiversity, new chemical structures may be helpful in the fight against malaria [5].

In this context, four surveys were performed between 2002 and 2004 on plants used in Cambodian traditional medicine. Extracts from 28 indigenous wild plant species were tested for their antiplasmodial activity. Among them, 26 extracts from eight plants were active against *P. falciparum. Stephania rotunda* extracts were selected for their interesting antiplasmodial activity [6]. Concentrations inhibiting 50% of parasitic growth (IC_50_) were respectively 1 and 2.8 μg/ml for dichloromethane and aqueous extracts of *S. rotunda* tuber on the *P. falciparum* chloroquine-resistant strain W2 [6]. *Stephania rotunda* (Menispermaceae) is a plant growing on calcareous cliffs and rainforest areas [7], used in traditional medicine to treat malaria and fevers [8].

Bioguided fractionation of dichloromethane extracts allowed the isolation of numerous alkaloids. Among them, a bisbenzylisoquinoline called cepharanthine (CEP) was one of the most interesting compound on W2 strain with an IC_50_ above 0.60 μM [9]. Moreover, it was tested in the BALB/c mouse infected by *Plasmodium berghei* ANKA strain. A dose of 10 mg/kg was administered by intraperitoneal and oral routes, and induced a decrease of parasitaemia by 47 and 50%, respectively [10].

A previous study reported a synergy between CEP and chloroquine (CQ) on the W2 strain [10]. According to Haruki *et al*., CEP is able to potentiate chloroquine on both sensible and resistant strains at low concentration (200 nM) [11], whereas Tamez *et al.*[12] showed that CEP was synergistic with CQ only on the resistant strain W2. CEP was reported to overcome multidrug resistance observed for anticancer drugs, by binding to phosphatidylserine in the plasma membrane and by perturbing membrane function [13]. CEP could interact with *pfmdr1* to decrease the effective dose of CQ, but it may not be associated with *pfcrt* since it does not reduce the IC_50_ to the level of the sensitive clone [12].

The use of monotherapy is no longer advisable in the treatment of malaria [2]. Thus, the aim of this paper was to evaluate the interest of CEP-based combinations. Several anti-malarial drugs were evaluated by isobolographic analysis. The more relevant synergic combinations were assessed in a malaria rodent model. In this work, it was decided to perform the study in the BALB/c mouse infected by *P. berghei* ANKA strain. Indeed, this mouse strain seems to die because of anaemia and hyperparasitaemia and to be resistant to experimental cerebral malaria induced by *P. berghei* ANKA line in many species [14,15]. The treatment consisted in the administration of CQ or amodiaquine (AQ) with and without CEP. This paper describes for the first time gain in life expectancy based on CEP in combinations.

## Methods

### *Plasmodium falciparum* culture

The culture-adapted reference strain of *P. falciparum* type W2 (Malaria Research and Reference Reagent Resource Center, MR4) came from Vietnam and is resistant to CQ, pyrimethamine and proguanil. W2 was maintained in culture in RPMI 1640 medium (Invitrogen, Paisley, UK) supplemented with 10% human serum (Abcys S A, Paris, France) and buffered with 25 mM HEPES-25 mM NaHCO_3_. The parasites were grown in type A^+^ human blood under controlled atmospheric conditions (10% O_2_, 5% CO_2_ and 85% N_2_) at 37°C with 95% humidity. All strains were twice synchronized with D-sorbitol 5% (Fluka, Saint Quentin Fallavier, France) [16].

### *In vitro* assay

CEP was isolated from *S. rotunda* tuber in the Pharmacognosy Laboratory (Aix-Marseille University, France) [17]. CQ, AQ, lumefantrine (LUM), atovaquone (ATO), piperaquine (PPQ), monodesethylamodiaquine (MdAQ), dihydroartemisinin (DHA) and mefloquine (MQ) were purchased from Sigma-Aldrich (St Louis, MO, USA).

The IC_50_ were evaluated with the Desjardins method [18] using tritied hypoxanthine. The specific activity of tritiated hypoxanthine is 1 mCi/mL (Perkin-Elmer, Courtaboeuf, France). The IC_50_ values were evaluated by analyzing incorporation of tritiated hypoxanthine according to the concentration by a non-linear regression analysis processing on dose–response curves (RiaSmart, Packard, Meriden, USA). Results were explained in geometrical average of IC_50_.

During this experiment, CEP was associated with DHA, MQ, ATO, PPQ, CQ, LUM, and MdAQ, following the fixed-ratio method of Fivelman *et al.*[19]. All compounds were diluted in methanol-dimethylsulphoxide (99:1, v/v) to prepare stock solutions at a concentration corresponding to eight IC_50_. Two molecules A and B were mixed within the ratios 5:0; 4:1; 3:2; 2:3; 1:4; 0:5. Two-fold serial dilutions of each stock solution were then prepared. Twenty-five μL of each solution were placed into 96-well plates in triplicate and left drying for one night. Then, 200 μL of a synchronous parasitized red blood (pRBC) cells suspension (final parasitaemia of 0.8% and final haematocrit of 2%) containing 40 μCi/mL of tritiated hypoxanthine were added.

### *In vivo* assay

Solutol® HS 15 was purchased from Sigma-Aldrich (St Louis, MO, USA). The BALB/c female mice were four weeks old and pathogens free (Charles River Laboratories, France). The *P. berghei* ANKA line was graciously provided Dr Salah Méchéri (Institut Pasteur, Paris, France).

The BALB/c mice were housed under standard conditions, with unlimited access to food and water. All experiments adhered to the French guidelines for animal research and were approved by the ethical committee of the Institut de Recherche Biomédicale des Armées - Antenne de Marseille (Number 2007–02). All efforts were made to minimize animal suffering.

Mice were infected with *P. berghei* ANKA parasites by intraperitoneal (ip) inoculation with 3.10^6^ pRBC from infected donor mice, diluted in normal saline. Blood drops were regularly taken from the tail veins to monitored parasitaemia with blood smears. The treatment was started when the parasitaemia of receive mice was positive. Drug were diluted in a 30% Solutol® HS 15 solution and then half diluted in water for injection.

Five groups of five or eight mice with average body weight of 16.7 g were randomly distributed. The control group received 75 μL (100 μL for 20 g of body weight) of the solvent and other groups were injected with 75 μL of molecules alone or in combination, during five days at the same time. In a first experiment, CQ was administered at the dose of 30 mg/kg alone or combined with 63 mg/kg of CEP (n = 5 per group). In a second experiment, AQ was administered at the dose of 10 mg/kg alone or combined with 21 mg/kg CEP (n = 8 per group). The parasitic growth was evaluated by blood smears. Mice were monitored until the death of all the mice for which a parasitic recrudescence was observed after the end of drugs administration. These delays were 32 and 71 days for CQ and AQ, respectively.

## Data analysis

### *In vitro* interactions

Solutions of combined drugs were two-fold serially diluted to generate a range of six concentrations in each case. Standard antiplasmodial assay conditions were then followed to provide a dose response curve. The assessment of drug interaction was based on the calculation of the fractional inhibitory concentration (FIC) of the two molecules (A and B). FIC was calculated for each association by dividing the IC_50_ of the drug in the combination by the IC_50_ of the drug alone. The sum of these two FIC (∑FIC) was calculated to plot isobologram curves [19,20] using the software R [21]. A straight diagonal line (∑FIC = 1) indicates an additive effect between drug A and drug B, concave curve (∑FIC < 1) indicates a synergistic effect and a convex curve (∑FIC > 1) indicates antagonism.

### Parasite multiplication rate

The parasite multiplication rate (PMR) is a good marker of parasitic death and allows a standardization of the results. The PMR is useful to normalize the activity of the molecules studied on parasite growth (treated), to the natural parasite growth (control). Indeed, it takes into account the initial parasitaemia of the infected mice when the treatment is started [22]. At each time a parasite ratio (PR) was calculated according to the formula:

PR=parasitaemiaattimeT/parasitaemiaattimezeroT0

The decrease in the rate of parasitic multiplication was then calculated with the formula:

PMR=PRtreated/PRcontrol

In the study presented here, the PMR were calculated over a period of 120 hours.

### Delay for parasitic recrudescence

Delay for parasitic recrudescence corresponds to the probability to record a positive parasitaemia at a given time after achievement of the treatment. The curves were fitted using GraphPad Prism® software version 5.04 (GraphPad Software Inc, 2012, La Jolla, CA, *http://www.graphpad.com/prism *).

### Survival curves

Survival curves were used to study the likelihood of death in treated and control mice. The survival analysis was performed according the method of Kaplan-Meier [23], which determines the probability of survival when at least one “death” is recorded. The survival curves were fitted using GraphPad Prism® software.

### Statistical tests

A log-rank test was performed to compare the survival and the delay for parasitic recrudescence curves. A two-ways analysis of variance (ANOVA) on treatment and time parameters was performed to compare PMR results, using the R software [21].

## Results and discussion

### *In vitro* assay

Previous susceptibility study of *P. falciparum* toward CEP was performed on the W2 clone. Thus, this clone was selected here for data comparison. The IC_50_ levels for the drugs alone were 1.26 ± 0.08, 1.62 ± 0.07, 9.86 ± 0.73, 20.3 ± 1.5, 42.9 ± 1.8, 58.9 ± 3.7, 710 ± 34 and 927 ± 65nM, respectively, for ATO, DHA, PPQ, LUM, MQ, MdAQ, CQ and CEP. *In vitro* susceptibilities to standard anti-malarial drugs as well to CEP were closed to those observed in the literature [10,24,25].

**Figure 1:**
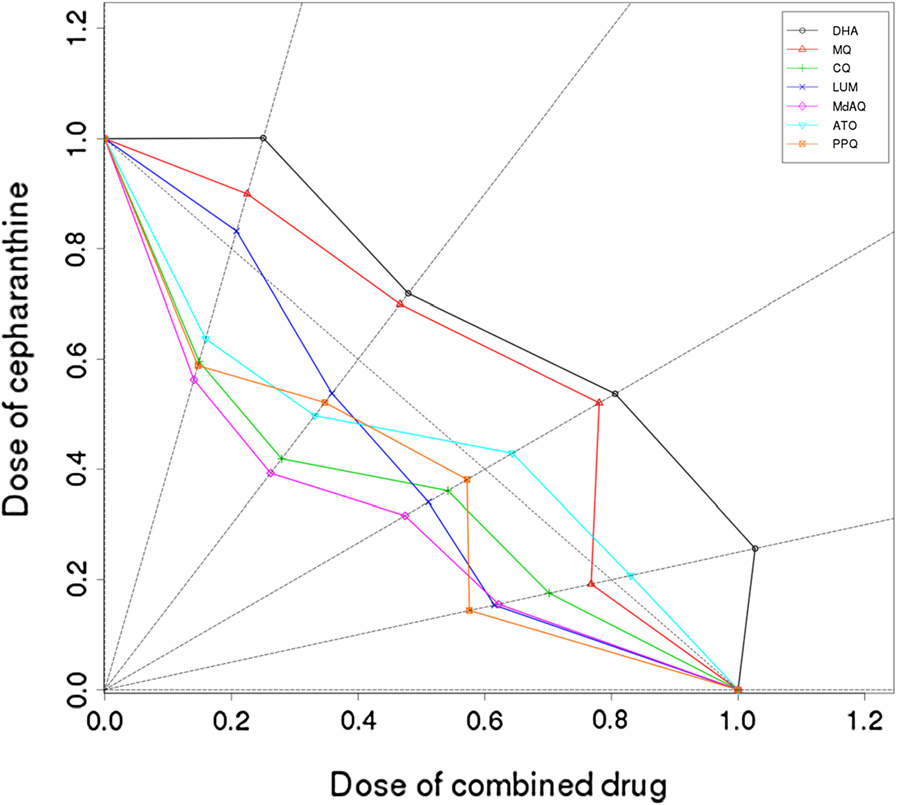
**Isobolograms obtained from the drug combination assays.** Percentages of CEP were plotted on the vertical axis and percentages of other combined molecules were plotted on the horizontal axis. Sums of FICs were calculated for each combination and reported on the corresponding straight line. When the sum is >1, the curve is convex and molecules trend toward antagonism. When the sum is <1, the curve is concave and molecules trend toward synergism. When the sum is = 1, molecules are additive. R is used to draw these curves.

The isobologram analysis of the drugs in various combinations is shown in Figure 1. The straight line with y-intercept and slope equal to 1 and -1 respectively is indicative of an additive effect whatever the drugs ratio. When CEP was combined with ATO, CQ and PPQ the ∑FIC were always below 1. Concave curves obtained with these three drugs are evidence of a synergistic interaction with CEP. Depending on the drugs ratio, interactions of CEP were synergic and additive with both LUM and ATO. By contrast, convex curves obtained by combination of CEP with DHA and MQ are evidence of an antagonistic interaction.

### *In vivo* assay

The presented results were obtained during two periods of experimentation. In a first one, combination of cepharanthine with chloroquine (n = 5 per group) was tested. In a second step, combination of cepharanthine with amodiaquine (n = 8 per group) was tested. As there was no statistical difference between the controls of the two experiments, data control have been merged to clarify the presentation of the results.

**Figure 2:**
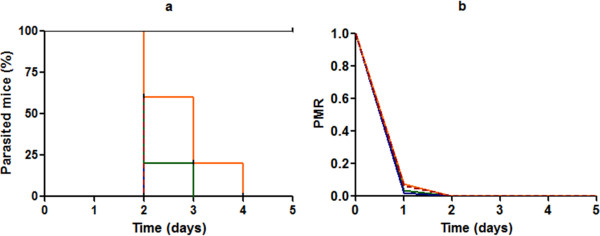
**Evolution of parasitaemia in Balb/c mouse. a**: Per cent of parasitized mice during the treatment. **b**: Parasitic multiplication rate. The blue straight line corresponds to the treatments with AQ alone (n = 8) and the green straight line corresponds to the treatments with CQ alone (n = 5). The red dotted line and the orange straight line correspond respectively to CQ (n = 5) and AQ associated with CEP (n = 8). The black curve represents the control mice (n = 13).

The CQ-sensitive *P. berghei* ANKA line may be used to generate experimental cerebral malaria. However, in BALB/c mice this ability seems to be absent and a very high mortality from anaemia and hyperparasitaemia is observed [14,15]. After inoculation, all the mice showed positive parasitaemia. When drug administrations were started, an average parasitaemia of 1.6% was observed (95% confidence interval, 1.2-2.0%). Figure 2 shows the percentage of infected mice as function as time curves in each group (a) in parallel to the PMR curves (b). Blood smears in the control group remained positive for all the mice whereas negative blood smears were observed for each mouse in each treated group in the range 48 and 96 hours after the first administration. A two-ways ANOVA analysis performed on the PMR curves, including time and treatment parameters, did not show significant differences between CQ or AQ alone and associated with CEP (p = 0.976 and 0.920, respectively). In contrast, benefits of combination-based CEP were observed with the two anti-malarial drugs, both on the survival (CQ: p = 0.0044 ; AQ: p = 0.0408) and parasites recrudescence curves (CQ: p = 0.0044; AQ: p = 0.0011). While blood smears were negative, a parasitic load may persist. A number of infected red blood cells below the limit of quantification of blood smear and/or a sequestration of parasite in the deep vascular compartment may explain the relapse which was observed. Thus, the description of the post-treatment phase is important to compare model susceptibility to the administered drugs.

**Figure 3:**
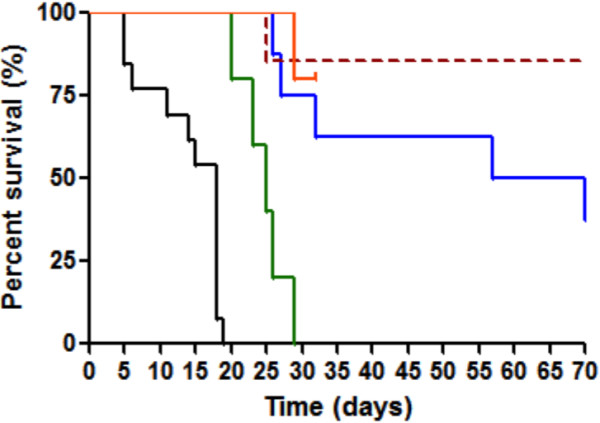
**Survival curves.** GraphPad Prism® is used to draw these curves. The colours used in this graph are the same as those used on Figure 2.

The survival curves (Figure 3) showed that all the treatments extended life expectancy in comparison with the control group (p <0.0001 for AQ ± CEP and p = 0.0004 for CQ ± CEP). When CQ was used alone, all the mice died before the 30^th^ day. Whereas, when CQ was associated with CEP, only one mouse died on the 28^th^ day. Thus, the combination of CEP with CQ significantly improved the life expectancy compared with CQ alone (p = 0.0044). Similar results were obtained with AQ (p = 0.0408). When AQ was administered alone, 75% (6/8) of mice died between the 20^th^ and the 71^st^ day of experiment. Whereas only one death was observed on the same period with the combination.

**Figure 4:**
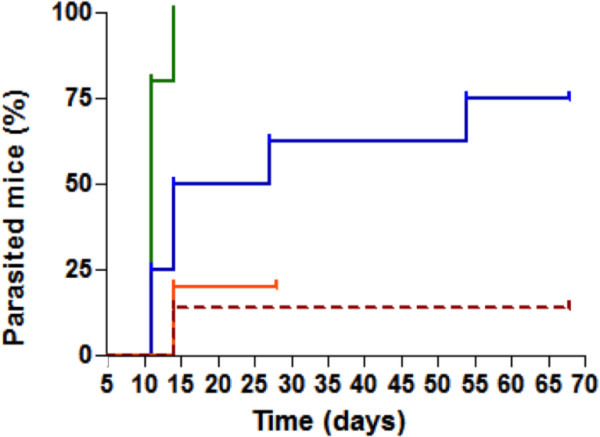
**Delay for parasitic recrudescence.** GraphPad Prism® is used to draw these curves. The colours used in this graph are the same as those used on Figure 2.

It is interesting to observe that the prolongation of the clinical follow-up highlighted the benefits of CEP in the dynamic of parasitaemia recrudescence. Indeed, for each treated group, a positive parasitaemia was once again observed few days after the end of drug administrations. This phenomenon is represented in Figure 4. The delay for recrudescence was statistically different between CQ or AQ alone and associated with CEP (p = 0.0047 and p = 0.0011, respectively). The two combinations more intensively delayed the parasitic growth compared with the use of molecules alone. Thus, CEP-based combinations impacted more intensively the parasitic load.

## Conclusions

In the battle against malaria, development of novel pharmacological approaches is paramount. Medicinal plants selected by ethnopharmacological approach are proving to be an important source of potentially therapeutic drugs. In this way, cepharanthine was isolated from extracts of *S. rotunda* and showed a potential original impact on the *Plasmodium* metabolism pathways. Indeed, it was been shown that CEP did not affect the crystallization of haem, the mitochondrial membrane potential and the production of free radicals [9]. Besides, the use of monotherapy must be abolished in the treatment of malaria to prevent the emergence of resistance to anti-malarial drugs. Thus, efficacy of some CEP-based combinations was evaluated in this work.

*In vitro,* synergic and additive effects were observed between CEP and ATO, CQ, LUM, MdAQ and PPQ. Among these potential combinations*,* CQ and its analogue AQ were chosen for further studies in the BALB/c mouse infected by *P. berghei* ANKA. The addition of CEP extended both the life expectancy of mice and the delay for parasitaemia recrudescence after the end of treatment in comparison with CQ and AQ alone. This shows that the impact on the parasitic load was higher with CEP-based combinations. The mechanism of this enhancing activity is not yet elucidated. Haruki *et al.*[11] suggested that CEP increases the access of CQ to ferriprotoporphyrin IX (FPIX) [11]. Regarding the synergistic effect observed with anti-malarial drugs, further studies should be performed with CEP alone and in combination on several *P. falciparum* strains and/or rodent malaria models to highlight its antiplasmodial activity.

## Abbreviations

ACT: Artemisinin combination therapy; ANOVA: Analysis of variance; AQ: Amodiaquine; ATO: Atovaquone; CEP: Cepharanthine; CQ: Chloroquine; DHA: Dihydroartemisinin; ECM: Experimental cerebral malaria; FIC: Fractional inhibitory concentration; FPIX: Ferriprotoporphyrin IX; IC50: Concentration inhibiting 50% of parasitic growth; LUM: Lumefantrine; MdAQ: Monodemethylamodiaquine; MQ: Mefloquine; PMR: Parasitic multiplicative rate; PPQ: Piperaquine; PR: Parasite ratio; pRBC: Parasitized red blood cell; WHO: World Health Organization.

## Competing interests

The authors have declared that they have no competing interests.

## Authors’ contributions

EO, DP and NT designed and coordinated the study. CD performed the *in vitro* study. CD and JD carried out the *in vivo* study. CD and NT analysed the data. Isobolograms were built by CC. CD and NT wrote the manuscript. All the authors read and approved the final manuscript.
